# Supplementary material for: A behavioral architecture for realistic simulations of Drosophila larva locomotion and foraging
Source: eLife. 2026 Aug 17;14:RP104262. doi: 10.7554/eLife.104262 (PMC13480992; doi:10.7554/eLife.104262)
Supplement: Peer review history

# Editor report: eLife Assessment

**DOI:** 10.7554/eLife.104262.3.sa0

**Authors:** Albert Cardona

**Affiliations:** University of Cambridge United Kingdom

**Keywords:** Convincing, Valuable

This **valuable** study presents a hierarchical computational model that integrates locomotion, navigation, and learning in *Drosophila* larvae. The evidence supporting the model is **convincing**, as it qualitatively replicates empirical behavioral data. While some simplifications in neuromechanical representation and sensory-motor integration are limiting factors, the reported modular framework will be of interest for computational modeling of biological movement and adaptive behavior.

# Referee report: Reviewer #1 (Public review)

**DOI:** 10.7554/eLife.104262.3.sa1

Summary:

The paper presents a three-layered hierarchical model for simulating Drosophila larva locomotion, navigation, and learning. The model consists of a basic locomotory layer that generates crawling and turning using a coupled-oscillator framework, incorporating intermittency in movement through alternating runs and pauses. The intermediate layer enables navigation by allowing larvae to actively sense and respond to odor gradients, facilitating chemotaxis. The adaptive learning layer integrates a spiking neural network model of the Mushroom Body, simulating associative learning where larvae modify their behavior based on past experiences. The model is validated through simulations of free exploration, chemotaxis, and odor preference learning, demonstrating close agreement with empirical behavioral data. This modular framework provides a valuable advance for modeling of larva behavior.

Strengths:

Every modeling paper requires certain assumptions and abstractions. The main strength of this paper lies in its modular and hierarchical approach to modeling behavior, making connections to influential theories of motor control in the brain. The authors also provide a convincing discussion of the experimental evidence supporting their layered behavioral architecture. This abstraction is valuable, offering researchers a useful conceptual framework and marking a significant step forward in the field. Connections to empirical larval movement are another major strength.

Weaknesses:

While the model represents a conceptual advance in the field, some of its assumptions and choices fall behind state-of-the-art approaches. One limitation is the paper's simplified representation of larval neuromechanics, in which the body is reduced to a two-segment structure with basic neural control. Another limitation is the absence of an explicit neuromuscular control system, which would better capture the role of segmental central pattern generators (CPGs) and neuronal circuits in regulating peristalsis and turning in Drosophila larvae. Many detailed neuromechanical models, as cited by the authors, have already been published. These abstractions overlook valuable experimental studies that detail segmental dynamics during crawling and the larval connectome.

The strength of the model could also be its weakness. The model follows a subsumption architecture, where low-level behaviors operate autonomously while higher layers modulate them. However, this approach may underestimate the complexity of real neural circuits, which likely exhibit more intricate feedback mechanisms between sensory input and motor execution.

# Referee report: Reviewer #2 (Public review)

**DOI:** 10.7554/eLife.104262.3.sa2

The paper proposes a hierarchically layer approach to larval locomotion, chemotaxis and learning. The model consists of a basic locomotor layer with two coupled oscillators, one for crawls and one for turns. The intermediate layer modulates the frequency and amplitude of tunings to enables chemotaxis. The higher layer, integrates a spiking neural network model of the Mushroom Body to modify the door valence in response to experience as during learning.

The model is compared to experimental data with a good degree of agreement. This modular framework provides a valuable advance for modeling larva behavior.

Strengths:

A novel multilayer level model that reflects current thinking of the neuronal organisation of motor control. The model is very useful to investigate the neuronal architecture of central pattern generators

and higher order motor control circuits that could be linked to larval connectome data.

Weaknesses:

All the limitations of the model are discussed and therefore the paper perfectly fits its purpose.

# Author comment: Author response

**DOI:** 10.7554/eLife.104262.3.sa3

**Authors:** Panagiotis Parthenios Sakagiannis, Anna-Maria Jürgensen, Martin Paul Nawrot

**Affiliations:** University of Cologne Cologne Germany; University of Cologne Cologne Germany; University of Cologne Cologne Germany

The following is the authors’ response to the original reviews.

**Public Reviews:**

**Reviewer #1 (Public review):**

We are happy to read that this reviewer considers the proposed behavioral architecture ‘a significant step forward in the field’, and that she/he recognizes the strengths of our work in the modular and hierarchical approach that provides connections to influential theories of motor control in the brain, in the experimental evidence it is based on, and in the valuable abstractions that we have chosen for the larval behavioral modeling.

The reviewer raises important points about the simplifications we have made, both conceptually and in the specific implementation of larval behaviors. Our main goal in this study is to introduce a conceptual framework that integrates agent-based modeling with systems neuroscience models in a modular fashion. To serve this purpose, we aimed for a minimal yet representative implementation at the motor layer of the architecture, calibrated to larval locomotion kinematics. This choice enables efficient simulation while allowing us to test top-down modulation and adaptive mechanisms in higher layers without the computational overhead of a full neuromechanical model. In addition to chemotaxis, we have recently used this simplified approach to model thermotaxis in larvae (Kafle et al., 2025, iScience, DOI: https://doi.org/10.1016/j.isci.2025.112809).

The reviewer notes the absence of explicit segmental neuromuscular control or central pattern generators (CPGs). We deliberately abstracted from these mechanisms, representing the larval body as two segments with basic kinematic control, to focus on reproducing overall locomotor patterns. This bisegmental simplification, which we illustrate in Supplemental Video “Bisegmental larva-body simplification”, retains the behavioral features relevant to our current aims. However, the modular structure of the framework means that more detailed neuromechanical models—incorporating CPG dynamics or connectome-derived circuit models—can be integrated in future work without altering the architecture as a whole.

We fully agree that real neural circuits are more complex than a strict subsumption architecture implies. In the Drosophila larva, there is clear evidence for ascending sensory feedback from the motor periphery to premotor and higher brain circuits, as well as neuromodulatory influences. These add layers of complexity beyond the predominantly descending control in our present model. At the same time, both larval and adult connectome data show that across-level descending and ascending connections are sparse compared to the dense within-layer connectivity. We see value in casting our model as a hierarchical control system precisely to make the strengths and limitations of such an abstraction explicit. The revised manuscript will include further discussion of these points.

In summary, our design choices reflect a trade-off: by limiting the biological detail in the lower layers, we gain computational efficiency and maintain a clear modular structure that can host models at different levels of abstraction. This ensures that the architecture remains both a tool for immediate behavioral simulation and a scaffold for integrating richer neural and biomechanical models as they become available.

**Reviewer #2 (Public review):**

We thank the reviewer for recognizing the novelty of our locomotory model, particularly the implementation of peristaltic strides based on our new analyses of empirical larval tracks, and for providing constructive feedback that will help us improve the manuscript.

The reviewer highlights the need for clearer explanations of the chemotaxis and odor preference modules. We expand these sections in the revised manuscript with more explicit descriptions of model structure, parameterization, and calibration. As mentioned above, we have also prepared a separate preprint dedicated to the *larvaworld* Python package, which contains detailed implementation notes and hands-on tutorials that allow users to adapt or extend individual modules.

Regarding the comparison to empirical behavior in chemotaxis, our present analysis is indeed primarily qualitative. However, we would like to emphasize that the temporal profile of odor concentration at the larval head in our simulations matches that measured in Gomez-Marin et al. (Nature Comm., 2011, DOI: https://doi.org/10.1038/ncomms1455) using only one additional free parameter, while all parameters of the basic locomotory model had been fitted to a separate exploration dataset before and were kept fixed in the chemotaxis experiments. In addition to the simulation of chemotaxis in the present paper, we recently used *larvaworld* in a practical model application to estimate a species-specific parameter of thermotaxis from experiments across different drosophilids (Kafle et al., 2025, iScience, DOI: https://doi.org/10.1016/j.isci.2025.112809).

The preference index in our simulations was computed using the same definition as in the established experimental group assay for larval memory retention, enabling a direct quantitative comparison between simulated and empirical results. Variability in the simulated outcomes arose naturally from inter-individual differences in body length and locomotory parameters, derived from real larval measurements, as well as from the random initial orientation of each individual in the arena. These factors contributed to variation in individual tracks and ultimately produced preference index values that closely matched those observed experimentally. In the revised manuscript, we also discuss handedness, as highlighted by the reviewer, as another meaningful expression of inter-individual variability in *Drosophila* larvae and insects more generally.

Finally, we acknowledge the reviewer’s concern about the scalability and broader applicability of the model. While the present paper focuses on three specific behavioral paradigms (exploration, chemotaxis, odor preference), the modular structure of the architecture is designed for flexibility: modules at any layer can be exchanged for more detailed or alternative implementations, and new sensory modalities or behaviors can be integrated without redesigning the system. *The larvaworld* package, associated codebase, and documentation are openly available to encourage adoption and adaptation by the larval research community.

**Reviewer #3 (Public review):**

This public review provides an excellent account of our central aim to build an easily configurable, well-documented platform for organism-scale behavioral simulation and we are happy to read that the reviewer considers this an excellent goal.

We thank the reviewer for her/his account of our well-organized code using contemporary Python tooling. We are currently further improving code readability and code documentation, and we will release a new version of the *larvaworld* Python package. We further agree with the reviewer’s assessment that understanding the model calibration currently requires reading of the appendix. For the revised manuscript we thus aim at improving our description of all calibration and modeling steps along the way. We will also make sure to improve the description of the experimental datasets used for calibration.

We recognize that our description of the paper’s scientific contribution could be clearer. In revision, we will sharpen the Introduction and Discussion to highlight our main contributions:

(1) Promoting a shift from isolated neural circuit modeling to integrated agent-based simulations in realistic environments.

(2) Proposing the layered behavioral architecture, adopting the subsumption paradigm for modular integration.

(3) Providing the *larvaworld* software as a ready-to-use, extensible modeling platform.

(4) Implementing an empirically calibrated locomotory model and demonstrating its integration with navigation and learning modules in replicated behavioral paradigms.

We agree with the reviewer that the next challenge is to integrate the empirically based behavioral simulations presented here with functional brain models capable of reproducing or predicting experimental findings at the level of cellular neurophysiology, including the effects of cell-type-specific manipulations such as gene knock-down or optogenetic activation/inhibition. However, based on our experience with systems-level modeling, we deliberately invested in behavioral simulation because functional models of the nervous system—including our own—often lack translation into simulated agent behavior. In many cases, model output is limited to one or more variables that can at best be interpreted as a behavioral bias, and most often represents an “average animal” that fails to capture inter-individual differences. By linking our spiking mushroom body model to behavioral simulations in a group of individual agents during memory retention tests (Figure 6C,D), we were able to achieve a first successful direct comparison between simulated and experimental behavior metrics—in this case, the behavioral preference index reported in Jürgensen et al. (iScience, 2024, DOI:https://doi.org/10.1016/j.isci.2023.108640).

Finally, we reiterate that the layered behavioral architecture is designed to promote a modular modeling paradigm. Our adoption of a subsumption architecture does not conflict with the concept of behavioral primitives; on the contrary, the notion that such primitives follow (semi-)autonomous motor programs and can be combined into more complex behaviors was the starting point for our implementation of the architecture in the fly larva. In our view, a genuinely contradictory paradigm for neural control of behavior would require a non-modular, strictly non-hierarchical organization of the nervous system and, by extension, of behavioral control.

**Recommendations for the authors:**

**Reviewer #1 (Recommendations for the authors):**

See public review for main points. To summarize, I find the conceptual framework of the paper very valuable and an important advance. However, in this age of data, I would have expected that the authors would make an effort to build more realistic models that could relate directly to neural data (including connectome and activity) and muscular dynamics at the segmental level.

This point is addressed in detail in our public review response. In brief, we agree that a segmental neuromechanical model informed by connectome data would provide richer mechanistic insight. However, such an approach would greatly increase complexity and reduce accessibility. Our aim here is to present a coarse-grained, kinematic-level framework that is modular, extensible, and designed to accommodate models at different levels of abstraction. Importantly, extensions that incorporate realistic neuromechanics or connectome-derived circuits can be readily integrated, provided they conform to the modular principles of the proposed behavioral architecture.

The authors do not cite figures in order or appearance, which makes it hard to read.

This has been corrected. Figures are now cited in the correct order throughout the revised manuscript.

I would explain the model in more detail in the main text. Currently, the model is introduced through Figure 1 in an abstract way. It is really hard to make the connection between this figure to the nuts-and-bolts of neuromechanics. And, I believe, for this paper, the details of the modeling matter and are not just technical points to be hidden in the appendix. The video (video 1) is not helpful.

We have restructured the Model section to provide more detail directly in the main text, moving explanations that were previously confined to the Appendix. This includes explicit description of the locomotory oscillator model, the intermittency module, and their empirical calibration. At the same time, we retained mathematical and implementation details in Materials & Methods to keep the reading flow accessible. Additionally, we expanded the caption of Video 1 and clarified in the text what it illustrates, making the video more informative.

Modeling choices lead to further weaknesses. While the model can replicate observed locomotory patterns, it does not fully explain the underlying neurobiological mechanisms that govern behavioral intermittency. For example, the crawl-bend interference mechanism, while capturing observed phase-dependent attenuation of turning, is implemented in a simplified, statistical manner rather than being derived from detailed neuromuscular dynamics. The intermittent locomotion model, which generates alternating runs and pauses, relies on log-normal distributed stridechains but does not explicitly model neural mechanisms responsible for switching between movement states.

We agree with this point. A fully mechanistic implementation of crawl-bend interference would require a detailed segmental neuromechanical model, which we deliberately refrained from integrating in order to keep the current study tractable and focused on a coarse-grained, kinematic-level description. Likewise, the intermittency module is currently based on data-fitted distributions of stridechains and pause durations, without explicit modeling of the neural mechanisms responsible for switching between these states. To our knowledge, these mechanisms remain unresolved, though alternative approaches have been suggested, for example, an artificial neural network model of intermittency (Sakagiannis et al., 2020). To ensure this limitation is transparent to the reader, we now explicitly state it in a newly added “Limitations of the study” subsection in the Discussion.

We also highlight that the behavioral architecture is designed to be extensible, so that future work may incorporate such mechanistic models when available, while preserving the modular framework.

I am curious about why the authors chose to model the mushroom body with much more realism than other modules.

We clarified that this choice was not due to a bias in modeling depth, but to demonstrate the modularity and flexibility of the architecture. The mushroom body (MB) model we integrated was developed in our previous work as a biologically realistic spiking neural network. By incorporating it into the current framework, we show that models of very different abstraction levels – from simple statistical oscillators to detailed spiking networks – can coexist and interact under the same architecture. This rationale is now explicitly stated in the Discussion.

**Reviewer #2 (Recommendations for the authors):**

The manuscript from Sakagiannis et al. proposes a novel model for locomotion and foraging in Drosophila. Their ambition is to make a unified model that will incorporate distinct layers of complexity to describe and predict the locomotor behaviour of a larva, during exploration, chemotaxis and even learning. The paper fails in doing so, starting with a rather interesting exploratory model and becoming less and less convincing as it progresses, with thinner (chemotaxis) and thinner (learning) experimental and theoretical support. The model for chemotaxis is extremely simplified compared to the work of other laboratories. The associative learning paradigm is taken from another paper from the same research group and is not sufficiently explained. In its current form, the paper is of very limited theoretical and practical value. The analysis is insufficient to judge the overall quality and scalability of the model. It is hard to know if the model could be adopted by others in the larval community more widely in other animals. Would it be flexible and robust enough to be used to model other behavioural conditions?

We appreciate this critical perspective. Our aim is not to present a final, fully parameterized model of all larval behaviors, but to introduce a flexible, modular behavioral architecture that integrates models at different levels of abstraction and can be expanded by the community. To support adoption, we have revised the manuscript to highlight the availability of the framework as a Python package (*larvaworld*), supplemented with documentation, tutorials, and code examples. This makes it easier for other researchers to reuse, extend, and test the architecture under additional behavioral conditions. We also explicitly refer to modeling studies that have adopted the proposed framework and the locomotory model itself.

Below, we address the reviewer’s points layer by layer.

(1) Exploratory behaviour. The strongest part of the paper. The authors propose a new method to analyse locomotion. They take into consideration the instantaneous linear and angular velocity. They assume the existence of two oscillators, which is really interesting. They incorporate the distribution of pauses duration and number of the strides. The incorporation of the strides is very exciting. They do not include handedness with has already been studied and incorporated in a mode for exploration they seem to have missed (Wosniack et al 2022). Figure 4 shows the dispersion. At first glance, it is very obvious that the model larvae do not behave like the animal. The distance they move from the centre is wider (Figure 4A). What is measured in dispersion (Figure 4B)? Just the distance travelled during 40s? A better measure of the similarities or differences between the model and real larvae would be interesting, such as analysing the Mean Square Displacement. Would the model be good if compared to the long-term exploratory behaviour from Sims et al. 2020, that the author previously used?

The authors should convince the readers that their model is better, or at least as good than the ones already available.

We thank the reviewer for these constructive suggestions. In the revised manuscript we now reference and discuss handedness, citing Wosniack et al. (2022, eLife), and highlight its potential role as an additional axis of individual variability. We also clarified the distance metrics used in Figure 4: dispersal denotes the Euclidean distance from the origin at the end of the trajectory, while pathlength denotes the cumulative distance travelled. Since larvae typically encounter the arena boundary within the first 40 seconds of exploration, dispersal is shown only over this interval.

With respect to the reviewer’s suggestion of using mean-squared displacement (MSD), we now explicitly describe the relation between dispersal and MSD. Dispersal is an individual-level displacement measure from which population-level metrics such as MSD can be directly derived.

Regarding long-term exploration, we agree that extended trajectories—as reported by Sims et al. (2020) over timescales of up to one hour—constitute a valuable complementary regime. Our experimental dataset is limited to 3-minute recordings in a bounded Petri dish, which constrains the accessible timescales of dispersal analysis. We now explicitly note in the Results that comparison to long-horizon datasets such as Sims et al. (2020) represents an important future direction that will require larger or unbounded arenas.

Together, these revisions strengthen the presentation of the exploration results and clarify how our model relates to established statistical measures of larval foraging behaviour.

(2) Chemotaxis. The chemotaxis model is so briefly explained in the result section that it is hard to understand. A modulation of the frequency and amplitude of lateral oscillator as a function of the concentration? The authors cannot differentiate between weathervaning and turning in this model (at least I can't understand how). What happened with the distribution of pauses and the directions of turns in Figure 5? The authors do not use real behavioural data to contract their model. How do we know that the parameters they have used reflect the larval behaviour? For example: what is the success rate for larvae to reach the area of high concentration? How close do they get? What is the length of the tracks from start to a target area of high concentration? Where are the calibration data for chemotaxis? This information is critical to understand the model, it needs to be shown in the result section. The authors mention an 8.9uM peak concentration. Of what?

The model is oversimplified in comparison with Davies et al. 2015 and it is not clear at all how it reflects the real chemotaxis, which is a rather complex behaviour.

We thank the reviewer for these detailed comments. In the revised manuscript we substantially expanded the description of the chemotaxis model. We now provide an explicit mathematical formulation of how odor concentration modulates the lateral oscillator through the quantity A_0_, which perturbs both the frequency and amplitude of bending according to the mechanism proposed by Wystrach et al. (2016). We additionally clarify that the motor layer - including the intermittency module and all parameters governing crawling, pausing, and turning - remains fully identical to the configuration calibrated on the exploration dataset; no refitting was performed for the chemotaxis condition.

To address the reviewer’s question regarding the distinction between weathervaning and head casting, we now explain that both behaviours emerge naturally from the same coupled-oscillator structure via stride-phase–dependent crawl–bend interference. High-amplitude headcasts occur during pauses when crawl-induced attenuation is lifted, whereas low-amplitude weathervaning arises during runs when the interference is active.

This unified mechanism eliminates the need for separate modules.

The chemotaxis experiments were implemented to qualitatively replicate the behavioural patterns described in Gómez-Marín et al. (2011, Fig. 1A–1F), and we now include explicit figure references in the captions. Because the present implementation is a proof of concept rather than a quantitatively calibrated chemotaxis model, we do not report success rates, approach distances, or track-length statistics, as these depend strongly on odorscape geometry and calibration against quantitative single-animal datasets that were not available for the current work. This clarification has been added to the text and is stated explicitly again in the Limitations section.

Finally, we now specify that the reported odor concentrations (e.g. 8.9,µM) follow the values used in Gómez-Marín et al. (2011), and we added the precise Gaussian function used to generate the odorscape in the Materials & Methods. Together, these revisions provide a clear and transparent account of the chemotaxis model and its scope.

(3) Associative learning paradigm. I assume that the authors intended to incorporate a bias in chemotaxis behaviour towards a particular odorant (CS) that would have been associated with a reward food (US). However the model works slightly differently, it is represented by an aversive and an appetitive gradient.

Theoretically, this is already an assumption (unless there is evidence for it, that should be referenced). It would be more conservative to have one neutral side and one appetitive (attractive) side. Second, the use of a mushroom body model, (even though it has already been published) to decide on the valence adds a layer of complexity that seems unnecessary. The learning process is different from the output process. Finally, the model intends to show us a "realist simulation of Drosophila locomotion" and we do not know how the larvae reach the right side during the test. It would be useful to have some comparison of the larval and model behaviour towards the preferred side.

In this last section, the objective of the research unweaves and falls short of its ambition.

We thank the reviewer for these helpful comments. In the revised manuscript we clarified that our implementation follows the standard larval conditioning protocol in which a rewarded odor (CS+) is tested against a neutral odor, not against an aversive one. The previously contradictory phrasing has been corrected, and the text now consistently reflects the established experimental procedure.

We further explain that the mushroom body (MB) model is included not in order to increase biological complexity in this section, but to demonstrate the flexibility of the proposed behavioral architecture: detailed circuit models and more abstract motor modules can coexist under the same framework. The MB model implements associative plasticity independently of any behavioral simulation, and its output - a scalar odor valence - is transformed linearly into an odor-gain parameter that modulates turning during the test phase. This separation between learning and behavioral output mirrors the logic of the biological system while keeping the overall architecture modular.

Regarding the reviewer’s request for insight into “how larvae reach the right side,” we note that standard group assays used in larval olfactory learning provide only population-level preference indices rather than detailed individual trajectories. Our comparison to empirical data therefore relies on these established preference indices, which the model successfully reproduces across training trials, including the characteristic saturation reported in Jürgensen et al. (2024). We now state explicitly that although the behavioral simulation does generate full trajectories for each virtual larva, the lack of corresponding experimental single-animal tracks precludes a direct trajectory-level comparison. This clarification has been added to the revised text.

Together, we believe that these revisions improve clarity and better situate the learning simulations within both the behavioral architecture framework and the constraints of available experimental data.

**Reviewer #3 (Recommendations for the authors):**

Figure 1a is very dense and I am struggling with the terms "reactive" and "basic" due to a general lack of clarity about the details of the model organization. For example, why do all of the sensory inputs point to turning proprioception? Why is proprioception two different things for turning and crawling? Why are some senses in light green while olfaction is in dark green? Why is feedback only from feeding, when crawling, head casting, and turning will change the sensory environment as well? Why is head casting not a behavioral module here? Why focus on following/being constrained by the "subsumption architecture paradigm" over a focus on the known literature and neuroanatomy?

We thank the reviewer for this careful inspection of Figure 1. In the revised version we improved both the figure and its caption, as well as the corresponding description in the text.

Specifically:

- The “basic” layer has been renamed the “motor” layer for clarity, and the caption has been expanded to better describe each component.

- The sensory inputs are now shown to target the motor layer as a whole, rather than just the proprioceptive component of turning.

- Each motor module is conceptualized as a sensorimotor loop (green-red), which explains why proprioception appears in both crawling and turning.

- The color coding has also been clarified: modules used in the current simulations are shown in darker shades, while others are faded.

- Sensory perturbations caused by body locomotion – as in the case of crawling and turning – are not depicted in the figure as feedback between modules. We make this more explicit in the caption. The signal from feeding to the above layers is neuromodulatory – as indicated by the purple arrowhead.

Finally, we explain that head casting and weathervaning are not modeled as separate modules, since both behaviors emerge from the coupled oscillator mechanism through crawl-bend interference. Our adherence to the subsumption architecture paradigm is motivated by its success in robotics and its conceptual alignment with hierarchical sensorimotor loops, but we have now made clearer that this is a simplifying framework rather than a rigid constraint.

"Stimulus free conditions" (line 102) don't really exist. Substrate and temperature will always be present, light will have some intensity, etc. Does this really refer to fictive behaviors?

We thank the reviewer for raising this point. In the revised manuscript we have removed the term “stimulus-free conditions” entirely to avoid the misleading implication that larvae experience no sensory input. We now explicitly describe these experiments as free exploration in the absence of navigation-guiding gradients, which accurately reflects the laboratory assay while avoiding any suggestion of fictive behavior. This terminology has been updated consistently throughout the text.

The first results section is closer to an introduction than the intro itself is, owing to its focus on the context of the work the paper actually does rather than a broad review of larval behaviors that are not considered within this work.

We believe the reviewer is referring to the “Model” section rather than the “Results.” The Model section is deliberately separated to outline the theoretical background of the behavioral architecture and to make explicit the general modeling assumptions, which explains why it cites previous work in detail. By contrast, the Introduction is intended as a brief overview of the broader larval behavioral repertoire, since the larva serves here as the case study for our framework. Presenting this repertoire is important because it defines the behaviors that populate the different layers of the architecture, even if only a subset of them is implemented in the simulations presented in this study.

While the model components are described in the modeling section, no question is actually discussed. What is the goal of this model?

This broader question is addressed in the public review section

"Crawler" and "turner" are inconsistently described. They are described as "modules" in Figure 1, but they seem more like behavioral primitives.

The specific terms "crawler" and "turner" refer to the computational modules, but correctly the reviewer points out that these generate the respective “crawling” and “turning” behavioral primitives. This has been made explicit in the Materials & Methods.

Do larva-larva interactions matter here?

In the revised manuscript we now state explicitly that larva–larva interactions are not included in the present simulations, as each virtual larva is modeled independently in accordance with the single-animal datasets used for calibration. We also point the reader to the Limitations section, where we note that although social interactions lie outside the scope of this study, the *Larvaworld* software package already supports tactile sensing and collision handling, enabling such interactions to be incorporated in future work.

The description of the locomotor system, with coupled oscillators between crawling frequency and bending is very empirical. Is this because of the 2-segment model effectively limiting peristalsis to a single segment? What are the limits of this approach?

The stride-phase–dependent modulation of bending amplitude was identified through kinematic analysis of full 12-segment larval datasets and is therefore independent of our later decision to implement a two-segment simplification. This means that the empirical relationship we describe should hold for any multisegment model, regardless of the reduced representation used in the present implementation. Generally, we performed our detailed empirical analyses with the goal to uncover statistical relations, which in turn were use for our data-driven coupled oscillator model in combination with the stochastic element of stride-chain and pause duration.

Line 190: The paper starts discussing experimental larva tracks. These experiments need to be described.

The reviewer probably refers to the dataset analysed in this study. This is a public dataset as described in the Dataset Description section in Materials & Methods, along with a description of the experiment per se.

The purpose of Figure 2 is not entirely clear. Several panels are not referenced in the text (F,G,H) and all panels are referenced extremely out of order. Figure 3 is similarly hard to follow for the same reasons of being referenced out of order. In fact, this section is largely duplicated by the "Model calibration" appendix, which I find to be much more clearly written and with more directly relevant figure panels.

In the revised manuscript, all panels of Figures 2 and 3 are now cited in the correct order, and their roles in the narrative have been clarified. Figure 2 is explicitly presented as a summary of the empirical kinematic analyses that motivate the structure of the locomotory model, while Figure 3 illustrates the corresponding model components. To avoid redundancy with the “Model calibration” appendix, we streamlined the main text and replaced duplicated descriptions with cross-references to the appendix, which contains the full methodological detail.

The data describe larvae behaving with a range of parameters, presumably both as individuals and across time. However, the models described seem to employ a population of larvae that shares a common best-fit parameter and the equations presented in the methods are all ordinary differential equations without noise or stochasticity. Where is the inter-individual variation coming from?

The reviewer is correct to point out the importance of variability. Our approach is agent-based, and we model populations of non-identical individuals rather than replicates of a single average larva. The simulated larvae retain variability across several parameters, capturing the combined range observed in the data. This was described in the original manuscript, and to avoid possible misunderstandings, we have now expanded the “Inter-individual variability” section in the Materials & Methods and, where appropriate, clarified this point elsewhere in the text.

The absolute orientation of trajectories in Figure 4A is not meaningful in your model. I suspect it would be more informative to show aligned trajectories in order to better visually assess the behavioral similarity. Also, the biological experiment needs to be described here. Time crawling seems to not be a great fit, although the peaks are fairly well aligned. Do you have thoughts on why this is?

In Figure 4A, which is intended as a visual comparison between experimental and simulated trajectories, the experimental tracks were transposed so that all starting points coincide at the center of the arena. As the reviewer notes, they were not rotated to a common axis, since our subsequent analysis focuses on spatial dispersal rather than directional alignment. The description of the experimental dataset has been clarified in the revised text.

The reviewer is also correct that the distribution of time spent crawling is narrower in the simulations than in the experimental data. This reflects the fact that in the present study only three crawling-related parameters were sampled to generate inter-individual variability, and time spent crawling was not among them. We deliberately chose to assess how well the model reproduces distributions for behavioral metrics that were not explicitly fitted or parameterized. This point has now been made explicit in the revised manuscript.

How did you assess the agreement of chemotaxis results with Gomez-Martin et al? It would be useful for the comparison to be made explicit within this paper, as well. How were the chemotaxis parameters fit?

The agreement between experimental and simulated chemotaxis was assessed only qualitatively, as we did not perform quantitative locomotor analyses on chemotaxis datasets. For these simulations we used the same motor layer, including all its modules, as calibrated in the free-exploration condition (Fig. 4). The only additional adjustment was a single weighting parameter that translates the appetitive or aversive valence of odor sources into modulatory input for the bending module. This parameter was tuned manually using a visual criterion of performance, to ensure that both attractive and aversive chemotaxis were observable. We now make explicit in the text that for more complex simulations we retain the calibration obtained in simpler conditions and build upon it, rather than re-optimizing the model. Moreover, we now provide reference to the exact figure numbers in Gomez-Martin et al. for direct visual comparison also of the perceived concentration metrics in our Figure 5E&F where experimental and simulated data show a very good correspondence.

Similarly, what are the key parameters for the mushroom body model and how did you fit their relationship to behavior? Was there actually feedback between the behavior of the larva and the training or was the SNN only used to generate the odor gain constant?

The reviewer is correct to highlight this point. In the present study the mushroom body model was simulated independently to generate the odor-specific behavioral bias. This output was then translated into an odor gain constant, which served as input for the subsequent behavioral simulations of odor preference. There was no closed-loop interaction between the larval behavior and the training of the spiking network in this version. Establishing such a closed-loop connection is part of our future goals.

It is unclear where feeding (as introduced in Figure 1) entered into the work presented, if at all.

The reviewer is correct that the feeding module does not play a role in the present study. It was included in the behavioral architecture for completeness and because it is already implemented in the *larvaworld* package (see Sakagiannis et al., 2024). We have clarified this in the revised text.

"During pauses, the input to the crawler module I_c = 0 and therefore forward..." The equations presented for the crawler module do not contain I_c.

The inconsistency regarding the crawler module input has also been corrected. The equations now explicitly include the tonic input parameter, making them consistent with the descriptive text and our model implementation.

Larva do more than crawl forward, they can also hunch up, head cast with their head in the air, dig, crawl backward, roll, and other behaviors. Because the individual modules in this framework have been defined as coupled oscillators, how would you decide to implement such aspects? At what point does the oscillator approach break down? In this model, how does the larva decide whether to bend left or right, and how is that affected by the environment or internal state? Can a larva bend in the same direction twice in a row?

The intermittent coupled-oscillator model presented here does not attempt to cover the full larval repertoire, such as hunching, digging, backward crawling, or rolling. Nor does it explicitly implement handedness as a directional bias. Nevertheless, the framework already allows for sequences of repeated turns: from a stationary position a larva can execute successive bends of varying amplitude, which may occur in the same direction, mimicking repeated head casts to one side.

Extending the model to include additional locomotor primitives would require the development of new modules, which could expand the basic locomotor layer either alongside or in place of the current lateral oscillator module. As noted in the manuscript, the modules implemented here are not intended as definitive but as placeholders that demonstrate how the architecture can integrate more elaborate models in the future. In this context, future directions include introducing handedness as part of inter-individual variability and enriching the behavioral repertoire with additional modules to capture the broader range of larval actions.

I was not able to install `larvaworld` either through pip in a fresh environment on OS X 15 and various Python versions between 3.8 and 3.12. I ran into a range of issues, from `tables` (which is understandable) to issues installing the old NumPy in Python 3.12 where `setuptools` is no longer included. The packaging should be made more robust, or the working environment could be better defined. For example, the version pinning of dependencies seems much more strict than I would expect for a user-focused Python library, particularly with out-of-date versions of core tools like NumPy.

We thank the reviewer for going to length and testing the implementation and pointing these issues to us. We have recently updated the package (version 2.0.1, November 2025) to improve installation robustness, relaxed unnecessary dependency pinning, and provided an environment specification to facilitate reproducibility. The revised manuscript directs users to recently updated installation instructions.

Automated testing for python versions 3.10-3.11 for MacOS, Windows and Ubuntu is already implemented. Unfortunately we have not yet tried it on OS X15. Please post any issues on the *larvaworld’s* github page : https://github.com/nawrotlab/larvaworld.
